# High p27 protein levels in chronic lymphocytic leukemia are associated to low Myc and Skp2 expression, confer resistance to apoptosis and antagonize Myc effects on cell cycle

**DOI:** 10.18632/oncotarget.2100

**Published:** 2014-06-27

**Authors:** Juan M. Caraballo, Juan C. Acosta, Miguel A. Cortés, Marta Albajar, M. Teresa Gómez-Casares, Ana Batlle-López, M. Angeles Cuadrado, Arantza Onaindia, Gabriel Bretones, Javier Llorca, Miguel A. Piris, Dolors Colomer, Javier León

**Affiliations:** ^1^ Instituto de Biomedicina y Biotecnología de Cantabria (IBBTEC), CSIC-Universidad de Cantabria-Sodercan, and Dpt. of. Biología Molecular, Universidad de Cantabria, Santander, Spain; ^2^ Hospital de Laredo, Laredo, Santander, Spain; ^3^ Servicio de Hematologia, Hospital Marqués de Valdecilla and Instituto de Investigación Marqués de Valdecilla (IDIVAL), Santander, Spain; ^4^ Servicio de Hematología, Hospital Dr. Negrin, Las Palmas, Spain; ^5^ Servicio de Anatomía Patológica, Hospital Marqués de Valdecilla and Instituto de Investigación Marqués de Valdecilla (IDIVAL), Santander, Spain; ^6^ Group of Epidemiology and Computational Biology, Universidad de Cantabria-IDIVAL, Santander, Spain and CIBER Epidemiología y Salud Pública (CIBERESP), Spain; ^7^ Institut d'Investigacions Biomèdiques August Pi i Sunyer (IDIBAPS), Hospital Clínic, Barcelona, Spain; ^8^ Present address: Edinburgh Cancer Research UK Centre, MRC Institute of Genetics and Molecular Medicine, University of Edinburgh, UK

**Keywords:** p27, Myc, Skp2, fludarabine, chronic lymphocytic leukemia

## Abstract

Myc (c-Myc) counteracts p27 effects, and low p27 usually correlates with high Myc expression in human cancer. However there is no information on the co-expression of both genes in chronic lymphocytic leukemia (CLL). We found a lack of correlation between RNA and protein levels of p27 and Myc in CLL cells, so we determined the protein levels by immunoblot in 107 cases of CLL. We observed a high p27 protein expression in CLL compared to normal B cells. Ectopic p27 expression in a CLL-derived cell line resulted in cell death resistance. Surprisingly, Myc expression was very low or undetectable in most CLL cases analyzed, with a clear correlation between high p27 and low Myc protein levels. This was associated with low Skp2 expression, which is consistent with the Skp2 role in p27 degradation and with *SKP2* being a Myc target gene. High Myc expression did not correlate with leukemia progression, despite that cell cycle-related Myc target genes were upregulated. However, biochemical analysis showed that the high p27 levels inhibited cyclin-Cdk complexes even in Myc expressing CLL cells. Our data suggest that the combination of high p27 and low Myc is a marker of CLL cells which is mediated by Skp2.

## INTRODUCTION

Chronic lymphocytic leukemia (CLL) is the most common leukemia in the Western countries and it is characterized by the progressive accumulation of clonal B lymphocytes in peripheral blood bone marrow and lymph nodes [1-3]. CLL is a heterogeneous disease with variable clinical presentation and evolution. Some patients have an indolent course with long survival without need for treatment while others experience an aggressive disease. Patients with no mutation in the variable region of the immunoglobulin genes (IGH) or with high expression of CD38 or ZAP70 had an aggressive course, whereas patients with mutated IGH clones or low expression of CD38 or ZAP70 cells usually show an indolent course [1, 3].

It has been reported alterations of cell cycle regulatory molecules in CLL, such as cyclin E, cyclin D, cyclin-dependent kinase (Cdk) 4 and Cdk2 [4, 5]. The Cdk inhibitor p27^KIP1^ (p27), that negatively controls cell-cycle progression, has been observed overexpressed in CLL cells [6-8]. This is in contrast to the majority of human tumors, where low levels of p27 are found [9-11]. p27 down-regulation in cancer has been associated with its function as an inhibitor of cell cycle. Indeed, a marked reduction in the abundance of p27 is common in many human tumors. In most cases where p27 regulation is described, the levels of p27 are mainly regulated at posttranslational level. This is mostly carried out by the SCF^Skp2^ ubiquitin ligase complex, where Skp2 acts as the p27-recogninzing subunit [12-14].

c-Myc (Myc herein after) is an oncogenic transcription factor or the helix-loop-helix/leucine zipper protein family. Myc forms dimers with the protein Max. These heterodimers bind to specific sequences called E-boxes in regulatory regions of target genes as well as intergenic regions. Myc-Max dimers bind to 15% of genomic loci and regulate about 1000 genes [15, 16]. Myc function integrating multiple signals, mediating transcriptional response that impinges on a wide array of biological functions such as cell cycle control, genomic instability, energetic metabolism, protein synthesis, intercellular communication and control of cell differentiation [17-22]. Consistent with these functions, high levels of this protein has been found in many human tumors [18, 23], and prominently in leukemia and lymphoma [24, 25].

In CLL, contradictory results on Myc mRNA levels in peripheral blood cells have been published. Some studies reported low Myc mRNA levels whereas high expression levels have been described in other [26-28]. *MYC* amplification and chromosomal rearrangements are very rare in CLL (less than 3%) but gains at 8q23.3-q24.3 (where *MYC* maps) was identified as a poor prognostic marker [29]. The frequency of *MYC* mutation, amplification and translocation increase in a subset of CLL with aggressive disease (30% of the cases) [30, 31] and in the CLL transformation to high grade lymphoma known as Richter syndrome [30, 32-34].

In cellular models, Myc blocks p27 antiproliferative activity and in most tumors there is an inverse correlation between Myc and p27 levels. Myc abrogates p27 function in proliferation arrest. This antagonism occurs through at least three levels. First, Myc represses p27 gene (*CDKN1B*) expression [35, 36]. Second, Myc induces cyclins and CDKs which can sequester p27 in CDK-Cyclin complexes [37-40]. Third, Myc induces the transcription of Skp2 [41]. The F-box protein Skp2 is the p27-recognizing subunit and the major responsible for p27 ubiquitination and degradation [42-44].

Despite their functional interactions, it is unknown whether Myc and p27 are coexpressed in CLL and whether Myc can compensate the reported high p27 expression. Here we studied the regulation of p27 and Myc in tumoral peripheral CLL cells and their correlation with the clinical features of the leukemia. In a cohort of more than 100 patients we analysed protein and mRNA expression of p27 and Myc. We found that p27 and Myc levels were inversely correlated, being p27 overexpressed and Myc downregulated. This correlation appears inverted in CLL with respect to conversely to most tumors. The excess in p27 counteracts Myc effect on cell cycle in the small number of samples with high Myc expression. We also found that low p27 and high Myc expression correlated with Skp2 suggesting a mechanistic explanation for Myc and p27 inverse correlation in CLL. Moreover, the enforced expression of p27 in a CLL-derived cell line resulted in resistance to apoptosis.

## RESULTS

### High p27 protein expression in CLL

**Figure 1:**
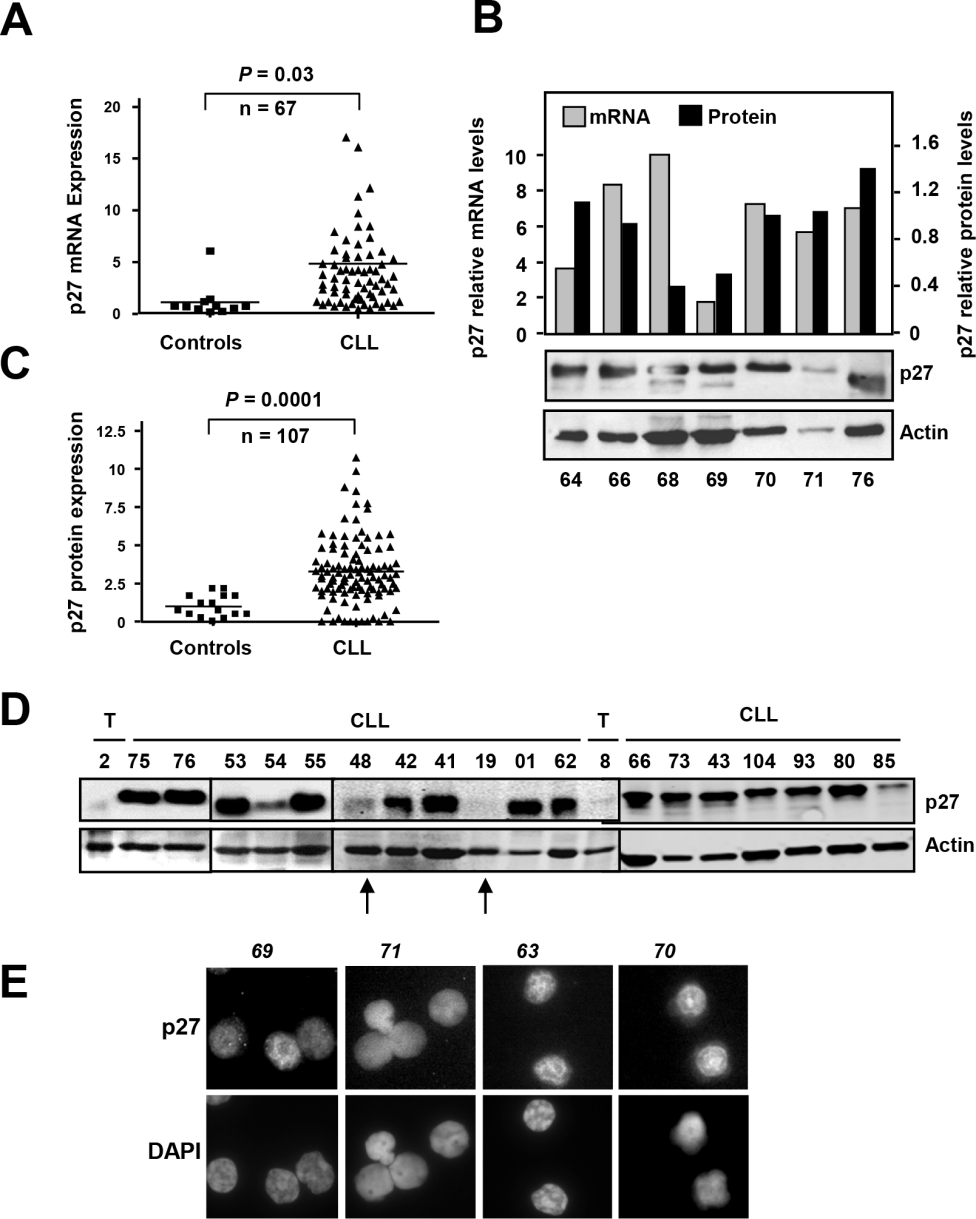
p27 expression in CLL samples **(A)** p27 mRNA expression in CLL cells and in healthy B-cells determined by RT-qPCR. **(B)** Comparison of p27 mRNA expression (determined by RT-qPCR) and p27 protein expression (determined by immunoblot) in the same CLL samples. The black bars show the densitometric quantification of p27 protein levels normalized to actin expression. **(C)** p27 protein expression in CLL cells and in healthy B-cells (controls). **(D)** A representative immunoblot showing p27 and actin expression. The arrows mark examples of CLL patient samples with low p27 expression. T, tonsils. **(E)** Immnofluorescence analysis showing nuclear p27 in CLL cells from four patients. Nuclei are stained with DAPI.

We first studied the mRNA levels of p27 in a cohort of 67 CLL patients by RT-qPCR or Northern blot analysis (Supplementary Table S1). The results revealed an increase (~5 fold as a mean) in p27 mRNA in CLL samples, as compared to controls (tonsil and peripheral blood B lymphocytes) (Figure 1A) The analysis of the mRNA data loaded in the Oncomine databank (www.oncomine.org) revealed a high heterogeneity among different studies. In some studies CLL samples showed higher levels of p27 mRNA with respect to controls, whereas these differences were not observed in other studies. Supplementary Figure S1 show two discordant studies. As previous results from our lab and others demonstrated an intense post-transcriptional regulation of p27 in human leukemia cells [45], we compared p27 mRNA (by RT-qPCR) and p27 protein (by immunoblot) levels in a subset of CLL cases. We confirmed the lack of correlation between both mRNA and protein levels in some samples (Figure 1B). In view of this result we decided to analyse p27 at the protein level in peripheral blood cells from 107 CLL cases by immunoblot. p27 signals were quantified by densitometry analysis and normalized against actin levels of each sample. The results showed that p27 protein is clearly overexpressed in CLL cells compared to normal B cells (86% of samples) (Figure 1C). A representative immunoblot showing p27 protein expression is shown in the Figure 1D. CLL cells showed a mean of 3-fold higher levels of p27 than controls. Only a minority of CLL samples showed levels of p27 protein similar to those observed in healthy control B cells. The high p27 levels in CLL cells is a striking finding because of the well-known role of p27 as inhibitor of the cell cycle progression. It is also in contrast to the situation in the rest of human tumors, where p27 is down-regulated, However, high p27 levels have been described in some tumors (breast, colon, melanoma, ovary, thyroid and lymphomas) where an aberrant cytoplasmic expression of p27 was observed [46-48]. Thus we explored the possibility of a p27 cytoplasmic localization in CLL cells. The immunofluorescence analysis revealed that p27 localization was mainly nuclear in the 9 fresh CLL samples analyzed. Some representative cases are shown in Figure 1E.

We next asked for a possible correlation of p27 protein expression and some CLL prognostic markers. We found that low p27 was associated to high ZAP70 expression and absence of 13q14 deletion (Supplementary Figure S2). No significant correlation was found in the others markers tested (expression of CD38, p53 deletion and ATM deletion), Furthermore we did not detect in our cohort of CLL patients a significant association of high p27 with Rai staging (*P* = 0.88, n = 85), progression of the leukemia (*P* = 0.69, n = 86) and with overall survival (*P* = 0.4, n = 86) (not shown).

### Low Myc protein expression in CLL

**Figure 2:**
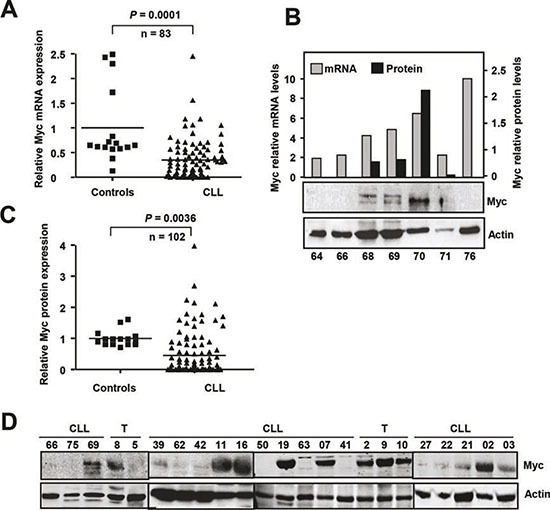
Myc expression in CLL cells **(A)** Myc mRNA expression in CLL cells and in healthy B-cells determined by RT-qPCR. **(B)** Comparison of Myc mRNA expression (determined by RT-qPCR) and p27 protein expression (determined by immunoblot) in the same CLL samples. The black bars show the densitometric quantification of Myc protein levels normalized to actin expression. **(C)** Myc protein expression in CLL cells and in healthy B-cells (controls). **(D)** Representative immunoblot showing Myc and actin expression. T, tonsils

In view of the involvement of Myc in B-cell malignancies and the Myc-p27 functional antagonism described in most models, we set out to examine Myc expression in our CLL cases (Supplementary Table S1). Myc mRNA levels were clearly down-regulated in CLL samples (n = 83) with respect to controls (Figure 2A). The Myc mRNA data loaded in the Oncomine databank (www.oncomine.org) revealed also contradictory results (two discordant studies are shown in Supplemental Figure S3). Our analysis revealed a poor correlation between Myc mRNA and protein levels (*P* = 0.39, n = 31) (Figure 2B). We analyzed Myc protein levels in 102 CLL samples by immunoblot and the Myc protein levels were quantified by densitometry and normalized against the actin levels. Most of patients showed undetectable or low levels of Myc protein, as compared to controls (Figure 2C, D). Only 18.6% of our samples (19 samples) showed a Myc expression higher than in control samples. It is noteworthy that only five patients (5% of cases) showed Myc levels ≥2-fold above control level. This low number of Myc-positive specimens makes it difficult to generate statistically significant data. However, we did not found any correlation between Myc expression and any of the bad prognosis markers analyzed (CD38 or ZAP70 expression, trisomy 12, ATM deletion, p53 deletion and 13q14 deletion). We also failed to detect a close correlation between high Myc protein levels and *NOTCH* mutation or overexpression (not shown). We did not detect a significant difference in the overall survival between Myc protein overexpressors and the rest of patients (*P* = 0.10, n = 82). Moreover we did not detect a significant impact of Myc protein levels on the progression of the disease (*P* = 0.15, n = 83). The main clinical characteristics of the patients with high Myc expression are summarized in the Supplementary Table S2.

**Figure 3:**
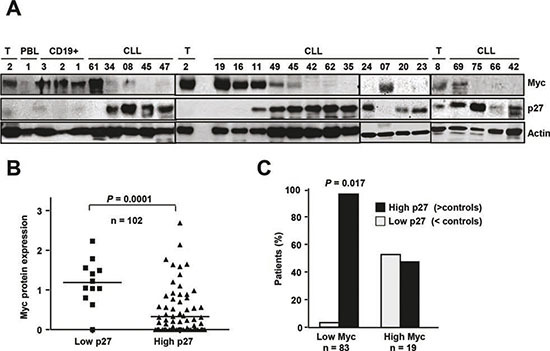
p27 and Myc coexpression in CLL cells **(A)** Representative immunoblot showing p27, Myc and actin expression in cells from CLL patients and from tonsil cells (T, tonsils). **(B)** Expression levels of Myc and p27 in CLL patients. The protein levels of p27 and Myc as determined by film densitometry and normalized to actin, were classified in three categories with respect to the mean of expression in controls. **(C)** Classification of patients with low and high Myc protein expression according to their p27 levels.

We next studied the expression of Myc and p27 in the same samples to explore the correlation between the Myc and p27 levels in CLL cells in a cohort of 102 CLL cases. Immunoblot studies revealed an inverse pattern of expression between p27 and Myc (Figure 3A shows a representative blot). The majority of the samples with low p27 expression showed high Myc levels (Figure 3B). The densitometric analysis of MYC signals in the blots revealed that most of the patients with low Myc expression (96%) showed high p27 expression (Figure 3B). Although it there was not a linear correlation between the levels of the two proteins, the Spearman's coefficient showed that Myc and p27 levels showed an inverse correlation (Spearman's Rho = −0.2047, *P* = 0.03, not shown).

Lymph nodes from CLL patients (n = 124) were also analyzed for the expression of p27 and Myc by immunohistochemistry. A marked nuclear p27 signal was observed in the non-proliferative center area of the node in all cases (representative micrographs are shown in Supplementary Figure S4). Myc was only expressed in the proliferative centers in a subset of nodes. Only 16% of the nodes showed expression of Myc in the 10% or more of the cells. Similarly to peripheral blood cells, there was no correlation between high Myc expression and Rai stage or disease progression (i.e., requiring treatment).

### p27 induces Myc downregulation and protects from apoptosis in CLL-derived cells

**Figure 4:**
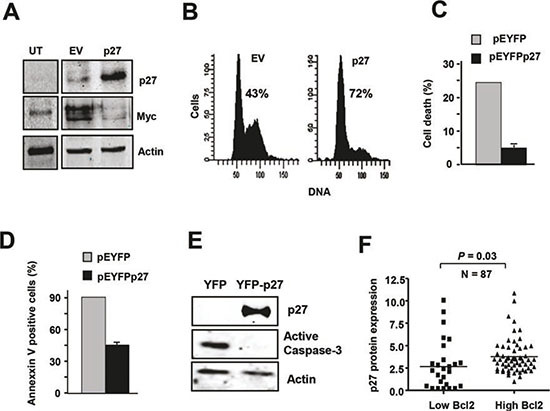
p27 and Myc are mutually regulated in MEC1 cells **(A)** Immunoblot showing p27 and Myc expression in MEC1 cell line transfected with a p27 expression vector (pCEFL-p27) and the corresponding empty vector. Proteins were analyzed 24 h after transfection. Actin levels are shown to asses protein loading. **(B)** MEC1 cells were transfected with pEYFPp27 vector and 24 h later the cell cycle of transfected cells was analysed by flow cytometry. The empty vector (pEYFP) was used as controls. The percentage of cells in the G0/G1 phase is indicated. **(C)** p27 expression rescued fludarabine-induced cell death. Cells were transfected with pEYFPp27 and pEYFP vector and 24 h after transfection the cells were treated with 10 μM fludarabine and cell death was determined 24 h later by trypan blue exclusion assay. **(D)** MEC1 cells were transfected and treated as in C, and the apoptosis was determined by annexin V binding, assessed by flow cytometry. **(E)** MEC1 cells were transfected and treated as in C and the levels of active caspase 3 were determined by immunoblot. **(F)** Correlation of high expression of p27 protein with higher Bcl2 levels in CLL cells.

To further investigate the mechanisms that could explain the striking expression pattern of Myc and p27 in CLL and their functional effects, we over-expressed p27 in the MEC1 cell line, a cell line derived from CLL cells. A p27 expression vector was transiently transfected into MEC1 cells, resulting in a dramatic increase in p27 levels with respect to empty vector-transfected cells (Figure 4A). Unlike peripheral blood CLL cells, proliferating MEC1 cells expressed high levels of Myc, but Myc expression was blunted by the over-expression of p27 (Figure 4A). We next tested the cell cycle profile of these p27-transfected cells to assess their functionality. As expected, p27-transfected cells were arrested in G0/G1 (Figure 4B). These results are consistent with the observations in vivo, where the majority of peripheral blood CLL cells are arrested in G0/G1, and high levels of p27 and low levels of Myc were observed. The gradual accumulation of immunologically dysfunctional B lymphocytes (most of them in G0 phase of the cell cycle) observed in CLL has been ascribed to defective apoptosis [49-51]. Therefore we explored whether p27 over-expression may also play a role in the resistance to apoptosis, a typical characteristic of CLL cells. For this purpose we generated a p27-YFP construct and transfected it into MEC1. As apoptotic stimulus we chose fludarabine, a drug commonly used in CLL treatment [52, 53] and which induces a decrease in p27 expression in CLL-derived cells in culture [5]. Apoptosis was determined after treatment of MEC1 cells transfected with the p27 expression vector with fludarabine. MEC1 cells with high p27 levels were more resistant to fludarabine-induced cell death than control cells as assessed by cell viability analysis using trypan blue (Figure 4C). We also measured the apoptosis in MEC1 p27-expressing cells by annexin V-binding by flow cytometry. The results showed that p27-expressing cells were more resistant to apoptosis (Figure 4D). The same result was observed by analyzing the expression of active caspase 3 (Figure 4E). Altogether these results show that p27 confers apoptosis resistance in MEC1 cells suggesting that the high p27 levels in CLL cells may contribute to the accumulation of leukemic cells. Overexpression of the anti-apoptotic protein Blc2 is a hallmark of CLL [54, 55] and therefore it may be expected a correlation between the p27 and Bcl2 levels in the CLL. We analyzed by immunoblot the expression of Bcl2 in our cohort of CLL samples and we found a direct correlation between Bcl2 and p27, showing that samples with low p27 expression also have low Bcl2 protein levels (Figure 4F).

### Cyclins and Skp2 regulation in Myc-expressing CLL cells

**Figure 5:**
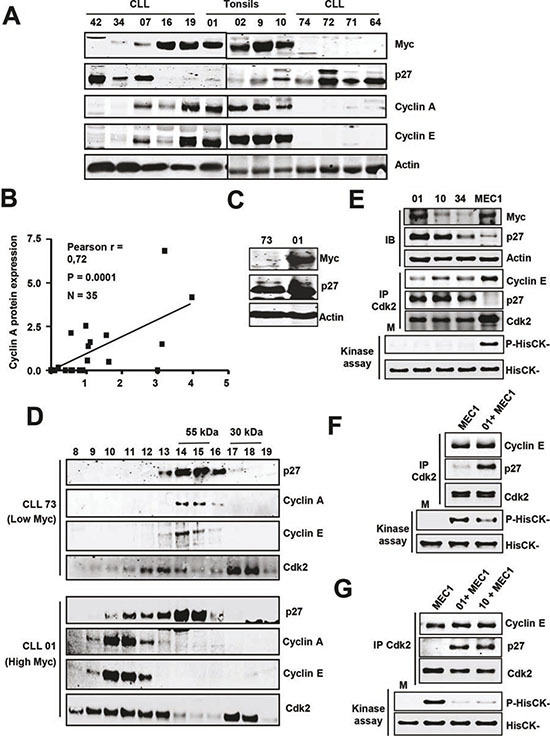
Expression of cell cycle regulators and cyclin-Cdk-p27 complexes in CLL cells **(A)** Representative immunoblot showing the levels of cyclins A and E in CLL cells with and without Myc/p27. Actin levels were also determined to assess protein loading. **(B)** Correlation between Myc and cyclin A protein levels in CLL cells. **(C)** Immunoblot showing Myc and p27 protein levels in four patient's cells selected for the experiments shown in D. **(D)** Molecular filtration chromatographic separation of CLL protein extracts followed by immunoblot for cyclins A, E and Cdk2. The elution of the 55 and 30 kDa proteins is shown at the top. **(E)** Immunoblot (IB) showing the levels of Myc and p27 in three CLL samples and MEC1 cells and kinase assay of Cdk2 in the same extracts. Proteins were immunoprecipitated with anti Cdk2 and the presence of both cyclin E, p27 and Cdk2 were determined by immunoblot. Lower panel: kinase assays were performed using HisCK^−^ as kinase substrate. M, mock kinase reaction without extract. **(F)** Immunoprecipitation of Cdk2 and kinase assay of the immunoprecipitates of MEC1 cells and MEC1 cells incubated with lysates from a CLL sample (#01). **(G)** Immunoprecipitation of Cdk2 and kinase assay from MEC1 cells and from mixed lysates prepared with MEC1 cells and two CLL samples (#01 and #10).

Given the role of Myc as an oncoprotein, the low Myc expression in most CLL cells was intriguing. We explored the possibility that perhaps Myc was not functional in these CLL cells. As Myc function as a heterodimer with the protein Max, we first asked for the expression of Max in our CLL samples. We showed that Max mRNA and protein were present in all samples analyzed (n = 78 and 26, respectively) and, interestingly, Max mRNA (Supplementary Figure S5A) and Max protein expression was higher in CLL than in controls (Supplementary Figure S5B). We next asked for the functionality of Myc in CLL cells expressing Myc. We first analyzed the expression of cyclins A and E, which are cell cycle regulators known to be induced by Myc [17, 18]. We selected three CLL cases with high and six with low Myc levels and the results clearly showed that in the cells with high Myc levels, protein expression of cyclin A and E were also higher (Figure 5A). Indeed, there was a clear correlation between Myc and cyclin A levels (Figure 5B).These results suggested that Myc is functional in CLL cells. Thus we next asked whether in CLL cells expressing both p27 and Myc, p27 was also functional, and can bind to and inhibit Cdk-cyclin complexes. Two approaches were used. First, the total lysate of B cells from two CLL cases expressing p27, one with high Myc levels and the other with low Myc levels (Figure 5C) were chromatographed through a gel filtration column on a FPLC apparatus. The results showed that in the presence of Myc there was a displacement of p27 from free forms into high-molecular weight fractions containing cyclins and Cdks complexes (Figure 5D), suggesting the formation of p27-Cdk-Cyclin complexes and thus the inhibition of Cdk2. To confirm the presence of these complexes we performed immunoprecipitations with anti-Cdk2 antibody and the results showed that p27 was bound to Cdk in Myc-expressing CLL cells (Figure 5E, upper panel). The previous results strongly suggested that p27 was inhibiting Cdk2 in CLL cells. To support this hypothesis, we directly determined Cdk2 activity in CLL lysates expressing both Myc and p27, as well as the ability of these lysates to inhibit Cdk2. First, we immunoprecipitated Cdk2 and its kinase activity was assayed by determining the level of phosphorylation at threonine 187 of recombinant inactive p27. The results show that the p27 present in the CLL cells was able to inhibit the endogenous Cdk activity, regardless the expression level of Myc (Figure 5E, lower panel). In a second approach we asked whether the p27 present in the CLL cells was able to inhibit exogenous Cdk2, using MEC1 extracts as the source of active Cdk2. This was performed in two ways. First we showed that lysates from p27-expressing CLL cells inhibited the Cdk2 purified by immunoprecipitation from MEC1 cells (Figure 5F). Second, we mixed lysates from MEC1 and two CLLs (with p27), and we showed that the immunoprecipitated Cdk2 from this mixtures was inactive, whereas the Cdk2 from MEC1 lysates was active (Figure 5G). This result is in line with the proposal that p27 overrides the effects of Myc as cell cycle stimulator, and are in line with the lack of a clear correlation between high Myc expression and progression of the leukemia. We conclude that, although Myc induces S-phase cyclins in CLL cells where it is expressed, the high p27 levels bind to most cyclin-Cdk complexes formed. Thus, the results suggest that the p27 present in the Myc-expressing cells would impair cell cycle progression.

**Figure 6:**
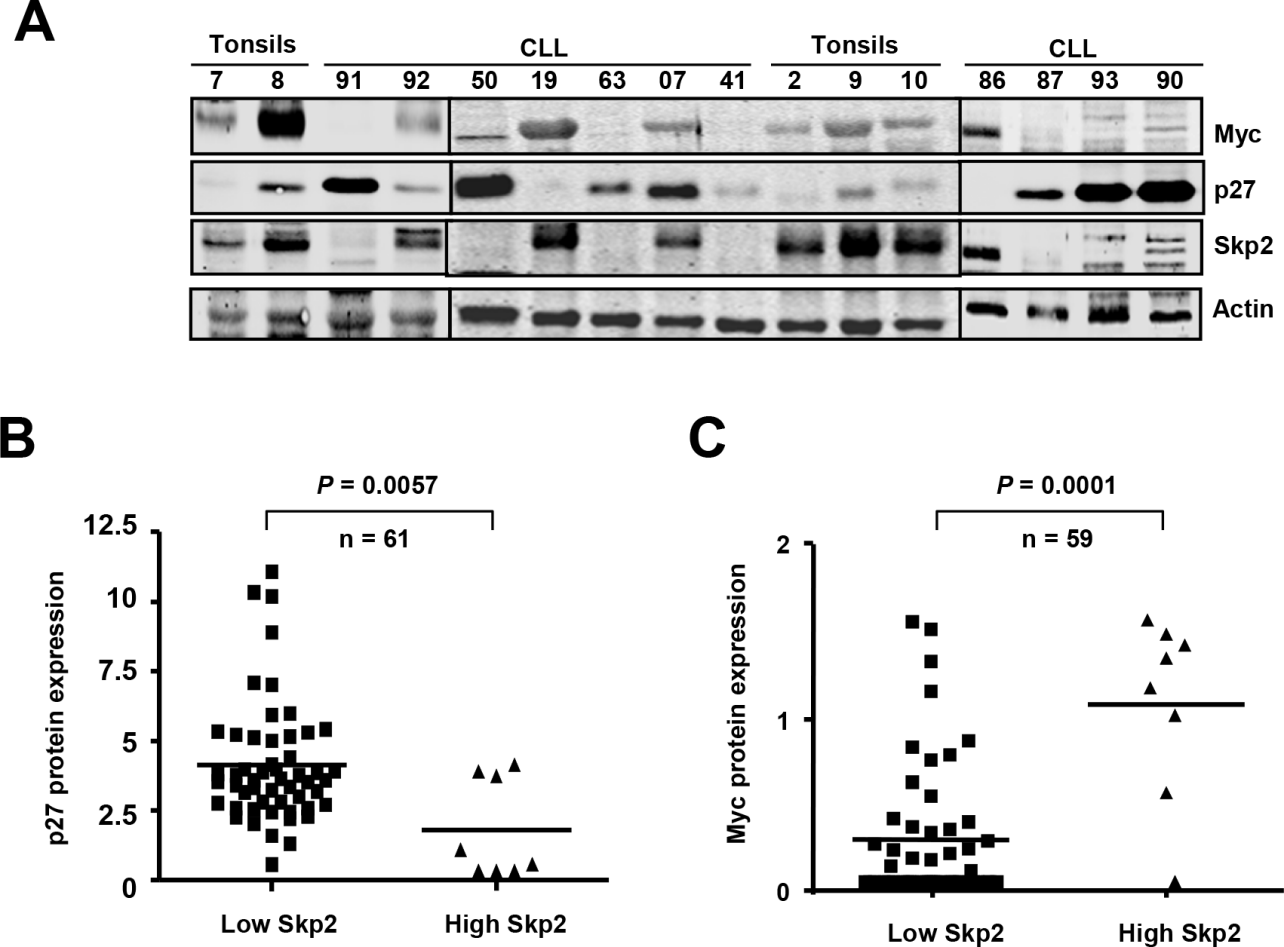
Overexpression of Skp2 in Myc-expressing CLL cells **(A)** Representative immunoblot showing the protein levels of Myc, p27 and Skp2 in CLL cells. Actin levels are also shown as protein loading control. **(B)** p27 protein levels in CLL cells with high or low expression of Skp2. **(C)** Myc protein levels in CLL cells with high or low expression of Skp2.

We were intrigued by the inverse correlation between Myc and p27 in CLL, which follows the opposite pattern than in most tumors. Samples with high levels of Myc (11%) (Figure 3B) show lower p27 levels, suggesting a mechanistic connection between the expression of both proteins. To explore this mechanism found in CLL we studied Skp2 expression. Skp2 is the main protein involved in p27 degradation and we previously reported that *SKP2* is a Myc-target gene [41]. Thus, we determined Skp2 protein expression, along with p27 and Myc, in our CLL cohort. The immunoblot results showed that high Skp2 levels correlated with high Myc and low p27 in almost all analyzed cases (a representative blot is shown in Figure 6A). In the samples with low Skp2 expression the mean levels of p27 protein were significantly higher (Figure 6B) whereas the mean levels of Myc protein were significantly higher in samples with high Skp2 (Figure 6C). The results argue for a pathway Myc-Skp2-p27 to explain the inverse correlation of Myc and p27 in CLL.

## DISCUSSION

It has been reported that expression of p27 has both prognostic and therapeutic implications in several tumors [9-11]. In sharp contrast to the scenery found in most or all human tumors including other leukemia we have found in CLL a high p27 expression and low Myc expression. We also found that p27 mRNA levels did not always correlate with p27 protein, which is in line with previous studies describing the intense posttranscriptional [8, 56, 57] and posttranslational regulation of p27 [10, 45, 58]. This argues against the relevance of mRNA-based studies. Therefore, we determined the p27 levels in 107 CLL samples by immunoblot and signal densitometry. The results showed a high p27 protein expression by immunoblot in a majority the CLL cases. These results agree with previous studies [6-8]. However, in contrast to previous reports [5], p27 was predominantly nuclear in our samples. High p27 expression has been reported to mark rapid progression of the disease [7] but we could not confirm these results in full as we we only observe a correlation between cases with ZAP70 expression and absence of 13q14 deletion with low p27 expression.

Why is p27 so highly expressed in CLL, despite the well-known activity of p27 as inhibitor of cell cycle progression? It is accepted that inherent defects in cell death of CLL lymphocytes are responsible for the accumulation of leukemic cells, the majority of them are arrested in G0 phase of the cell cycle [3, 49]. G0 arrest is consistent with the Cdk inhibition brought about by p27, particularly Cdk2. We hypothesize that p27 could contribute to the resistance to cell death of CLL cells, either as a consequence of the cell cycle arrest or through other less defined mechanisms. To explore this hypothesis we generated and transfected p27 expression vectors into the CLL-derived MEC1 cells, and we found that p27 overexpression resulted in resistance to apoptosis. The correlation beween high p27 and low apoptosis was also consistent by the correlation between p27 and Bcl2 expression that we found in CLL patients.

Myc is upregulated in leukemia and lymphoma [25] and Myc counteracts the p27-mediated inhibition of proliferation in many models. However, the correlation between Myc and p27 in CLL cells has never been analyzed. Previous studies have reported controversial data so as Myc mRNA expression in peripheral blood CLL cells [26-28, 59-61]. However, Myc protein stability is also under intense postransductional regulation [62-64] and, as in the case of p27, mRNA-base studies can offer misleading information so as the levels of Myc protein in CLL cells. Therefore we studied the Myc protein levels by immunoblot. The results showed a very low expression of Myc protein in CLL cells. Actually, in a 49% of our CLL samples, Myc was under our level of detection by immunoblot. Moreover in most of our patients classified as “Myc positive”, Myc levels were not higher than in normal tonsil B-cells. We observed the opposite correlation in CLL, i.e., high p27 and low Myc in the peripheral blood CLL lymphocytes of most patients (78%) whereas only 45% of the patients with p27 expressed Myc above the level detectable in our immunoblots. The low Myc and high p27 expression that we observed in a majority of CLL cases is the opposite pattern observed in most human tumors. It is noteworthy that in the small subset of CLL samples with high Myc expression we did not detect a significantly faster disease progression, despite that those cells show molecular hallmarks of Myc-transformed cells, i.e., high levels of cyclins A and E. Furthermore, in these Myc-expressing cells, p27 was found in complexes with Cdk-cyclins and Cdk2 kinase activity was efficiently inhibited. We hypothesize that in those cases with concomitant p27 and Myc expression, p27 overrides the activity of Myc-as cell cycle stimulator. The hypothesis is in line with the lack of a clear correlation between high Myc expression and progression of the leukemia.

We explored whether there is a mechanistic link between the low Myc and high p27 expression in CLL. Our results show very low levels of Skp2 in the majority of CLL cases, in correlation with low p27. Moreover, Skp2 mRNA and protein levels were elevated in those cases with high Myc expression. This is in full agreement with *SKP2* being a Myc target gene which expression in proliferating cells depends on Myc [41]. As Skp2 is a subunit of the SCF^SKP2^ complex that promotes p27 degradation [12, 13], Skp2 levels could explain the inverse correlation between Myc and p27 in CLL. Therefore, we suggest that in CLL cells Myc induces p27 degradation through up-regulation of Skp2 mRNA in CLL cells. Altogether, the results offer an explanation for the striking Myc and p27 expression pattern found in CLL. Further work is necessary to dissect out the contribution of the Myc-p27 axis to CLL pathogenesis and their use as markers of the disease.

## MATERIAL AND METHODS

### Patient samples and progression criteria

Peripheral blood lymphocytes from 159 CLL patients were studied at the mRNA level, protein level or both (Supplementary Table S1). CLL samples were obtained from the Hospital Universitario Marqués de Valdecilla of Santander (138 samples), Hospital Universitario Dr Negrín of Las Palmas (28 samples) and from Hospital Clínic of Barcelona (10 samples). Written informed consent in accordance with the Ethics Committee of each hospital and the Declaration of Helsinki were obtained. The biological characteristics of the patients (age, sex, clinical stage according to Rai classification, cytogenetic alterations, treatment and number of samples analyzed for RNA and protein) or 11q14) are summarized in Supplementary Table S1. Control samples were obtained from healthy donors (peripheral blood lymphocytes) and from tonsillectomy of patients with tonsillitis (tonsils). CLL cells were isolated by Ficoll-Paque or flow cytometry using anti CD19. Most of patients (60%) were diagnosed in an early stage of the disease (stages 0 and I). 43% of patients progressed, most of these (81%) required treatment. Progressive disease was considered when at least one of the following criteria was present: a) evidence of progressive marrow failure manifested by the development anemia (Hb <10g/dL) and/or thrombocytopenia (<100×10**^9^**/L); b) massive (i.e., at least 6 cm below the left costal margin) or progressive or symptomatic splenomegaly; c) massive nodes (i.e., at least 10 cm in longest diameter) or progressive or symptomatic lymphadenopathy; d) progressive clonal lymphocytosis with an increase of more than 50% over a 2-month period or lymphocyte doubling time of less than 6 months.; e) autoimmune cytopenia that is poorly responsive to standard therapy; f) constitutional symptoms. 124 lymph nodes of CLL with ganglionar affectation were also included in the study. Samples were obtained from the Biobank of the IDIVAL-Hospital Universitario Marques de Valdecilla. 16 samples of non-metastatic nodes in surgical specimens were used as controls.

### Analysis of leukemia markers

The percentage of tumoral cells (CD19+, CD5+) as well as the expression levels of ZAP70 and CD38 was analyzed by flow cytometry as follow. A hundred microliters of whole peripheral blood in EDTA were incubated with CD38-FITC (Citognos), CD19-PERP-Cy5, CD20-APC and CD5-PE-FITC (antibodies from Becton-Dickinson) to identify surface membrane antigens. Then cells were fixed and permeabilized using the Fix & Perm kit (Caltag Laboratories, Burlingame, CA) and incubated with the ZAP70 monoclonal antibody (R-phycoerythrin conjugated; clone 1E 7.2, Caltag Laboratories). After, cells were analyzed by flow cytometry (BD FACSCanto, Becton Dickinson Immunocytometry Systems, San Jose, CA). ZAP70 results were expressed as the percentage of CD5/CD19 positive cells compared to T cells (cut-off 20%). CD38 expression was defined as positive when identified in more than or equal to 30% of the gated CD19/CD5 positive cells. Cytogenetic alterations were assessed by fluorescence in situ hybridization (FISH) using the FISH LLC Multicolor Kit (LSI D13S319-13q14)/13q34/CEP12, LSI p53/LSI ATM) (Vysis-Abbott Molecular, USA) At least 100 intact, non-overlapping nuclei were analyzed. Control values were previously established based on samples of 10 controls X±3SD (mean plus three standard deviations). The cut-offs value for p53, 13q14 or ATM deletion was 10%, and for trisomy 12 was 3%.

### Cell culture and transfection

MEC1 cells (DSMZ-497), derive from human CLL in prolymphocytic transformation [65], were grown in RPMI 1640 medium supplemented with 10% fetal calf serum and antibiotics at 37°C and 5% CO_2_. MEC1 were transiently transfected by nucleofection (Amaxa electroporator) with pCEFL-p27, pEYFP-p27, pRS-shMyc or the corresponding empty vector. A green fluorescent protein vector (pmaxGFP, Amaxa) was co-transfected to assess transfection efficiency. pEYFP-p27 was constructed by inserting the human p27 cDNA into the BamHI and XbaI restriction sites of pEYFP-C1 vector (Clontech). A hemocytometer was used to count the concentration of cells. Dye exclusion method was used for assessment of cell viability. Trypan Blue was the vital stain used to selectively color dead cells. MEC1 transfected with a p27 expression vector and with empty vector were treated with 10 μM fludarabine (Sigma-Aldrich) for 24 hours.

### RNA analysis

RNA was extracted by Trizol or RNeasy kit (Qiagen). First-strand cDNA was synthesized from 1 μg of total RNA using Script reverse transcriptase (BioRad). Quantitative RT-PCR was performed with the SYBR Green PCR kit (BioRad). The expression levels were normalized to the expression of ribosomal protein RPS14 mRNA. The following primers were used: for Myc 5’- TCGGATTCTCTGCTCTCCTC-3’ and 5’- GAGCCTGCCTCTTTTCCAC3’; p27 5’- CCGGCTAAC TCTGAGGACAC-3’ and 5’- AGAAGAATCGTCGGT TGCAG-3’; RPS14: 5’- TATCACCGCCCTACACA TCA-3’ and 5’- GGGGTGACATCCTCAATCC-3’. The mean value of Myc and p27 mRNA from of 16 control samples (tonsils and CD19+ cells) were assigned value =1. mRNA levels of the CLL samples were normalized against this value Northern blot analysis for p27, Myc anc Max was performed as described previously [45, 66].

### Immunoblots

CLL and MEC1 cells were lysed in lysis buffer (1% NP40, 0.5% SDS, 50 mM Tris-HCl pH 7.5, 150 mM NaCl, 1 mM EDTA, 10% glycerol, 10 mM NaF and protease inhibitors) for 20 minutes at 4°C. Lysates were cleared by centrifugation. 40 μg of lysates were subjected to SDS-PAGE and immunoblot as described previously [45]. The antibodies used were anti-Actin (I-19, goat polyclonal, sc-1616), anti-MYC (N-262, sc-764), anti-SKP2 (H-435, sc-7164), anti-p27 (C-19, sc-528 and sc-528-G, rabbit and goat polyclonal respectively) anti cyclin E (M-20, sc-481) or anti-cyclin E (HE12, mouse monoclonal, sc-247), anti-cyclin A (H-432, sc-751), or anti-CDK2 (M2, sc-163) (unless otherwise indicated, all rabbit polyclonals from Santa Cruz Biotechnology), anti-p27 monoclonal antibody (K-25020; Transduction Labs), and anti-Thr(P)-187-p27 (rabbit polyclonal, 71-7700; Invitrogen). The blots were developed with secondary antibodies conjugated to IRDye680 and IRDye800 (Li-Cor Biosciences) and visualized in an Odyssey scanner. Immunoblots quantification and densitometry analysis were carried out using the ImageJ software. p27 and Myc values were normalized to the actin protein level in each sample. The mean value of Myc and p27 protein from of 15 control samples (tonsils and CD19+ cells) were assigned value =1. Proteins levels of the CLL samples were normalized against this value

### Immunoprecipitations and kinase assays

For immunoprecipitations, cells were lysed in non-denaturing lysis buffer (50 mM Tris pH7.5, 150 mM NaCl, 0,5% NP40, 1mM EDTA and protease inhibitor cocktail) and protein extracts were cleared by centrifugation. Protein extracts (1 mg per assay) were immunoprecipitated with 1 μg of anti-CDK2 (M2, Santa Cruz Biotechnology) and collected on protein G-Dynabeads (Invitrogen). After extensive washing with non-denaturing lysis buffer, immunocomplexes were subjected to SDS-PAGE and immunoblot analysis as previously described. For kinase assays, after washing with non-denaturing lysis buffer, immunocomplexes were additionally washed in kinase buffer (50 mM Hepes-NaOH pH7.2, 150 mM NaCl, 10 mM MgCl2, 2.5 mM EGTA, 1 mM EDTA, 1 mM dithiothreitol, 10% glycerol, 10 mM β-glycerophosphate and 10 mM NaF) and resuspended in kinase buffer (40 μl) supplemented with 50 μM ATP and 0.1 μg of recombinant His6-p27CK^−^ peptide (HisCK^−^), as described [67]. HisCK^−^ vector was constructed by subcloning the cDNA into the pET28a vector (Novagen), and the peptide was expressed in *E. coli* BL21 strain upon IPTG induction, and purified through Ni affinity columns (Macherey-Nagel). The kinase reaction was incubated for 60 min at 30°C and stopped by addition of Laemmli loading buffer to the reaction mixture. Samples were then heated at 95°C for 5 min and subjected to SDS-PAGE and immunoblot analysis with anti-p-Thr187-p27 (rabbit polyclonal, 71-7700, Invitrogen) first and with anti-p27 (Goat polyclonal, sc-528-G, Santa Cruz Biotech.) later. The signals were revealed with anti-rabbit and anti-goat secondary antibodies conjugated to IRDye680 and IRDye800, respectively (Li-Cor Biosciences). Immunoblots were scanned in an Odyssey scanner.

### Immunofluorescence and immunohistochemistry

Cytospin preparations were fixed with paraformaldehyde 3.7% in PBS for 10 minutes to room temperature and permeabilized with 0.2% triton X-100 (10 minutes). Anti-p27 (C-19) and anti-Myc antibodies (N-262, all antibodies were rabbit polyclonals from Santa Cruz Biotech.) were incubated overnight, and Texas Red or FITC-conjugated secondary antibody (Dako) were used to detect the presence of p27 and Myc. Samples were mounted with Vectashield (Vector) containing 4’-6-diamidino-2-phenylindole (DAPI) to stain nuclei and photographed under a fluorescence microscope. For immunohistochemistry, the CLL, sections of CLL lymph nodes were arrayed into a new paraffin block using a tissue microarray (TMA) workstation (Beecher Instruments, Silver Spring, MD). Immunohistochemical staining for Myc (Y29 rabbit polyclonal antibody from Dako) and p27 (SX53G8 monoclonal antibody fom Dako) was performed following conventional automated protocols in a Autostainer Plus device (Dako).

### Gel filtration chromatography

CLL cells were lysed in a 0.5% Triton X-100, 50 mM Hepes/NaOH pH 7.4, 150 mM NaCl, 1 mM EDTA, 2,5 mM EGTA, 1 mM dithiothreitol (DTT), 1 mM phenylmethylsulphonyl fluoride (PMSF), 10% glycerol (w/v). and 1/100 protease inhibitor cocktail (Calbiochem) and 2 mg protein samples in a volume of 200 μl were applied onto a Superdex 200 10/300 GL column (GE Healthcare) equilibrated with chromatography buffer (50 mM Hepes pH 7.4, 150 mM NaCl, 1 mM EDTA, 2,5 mM EGTA, 1 mM DTT, 1 mM PMSF and 10% glycerol) and subjected to fast-performance liquid chromatography in an ÄKTA apparatus (GE Healthcare) with a flow rate of 0.4 ml/min at 4°C. 500 μl fractions were collected and subjected to western blot or immunoprecipitation. Molecular mass standards for the gel filtration column were: apoferritin (443 kDa), catalase (232 kDa), BSA (66 kDa) and ovalbumin (45 kDa).

### Apoptosis assays

MEC1 were transfected with p27 and treated with fludarabine (Sigma). Percentage of death cells was determined by the Trypan Blue dye exclusion test, by annexin V binding detected by flow cytometry (kit of Immunostep Research) and by immunobloting with anti-active caspase 3 (Immunostep Research).

### Statistical analysis

Spearman's rank correlation, Pearson's correlation, *t*-test and distribution were used to determine correlations, dependence and statistical significance. Survival curves were analyzed according to the Kaplan and Meier method. SPSS Statistics 17.0 and GraphPad Prism software were used to different analysis.

## SUPPLEMENTARY MATERIALS TABLES AND FIGURES


