# Supplementary material for: High p27 protein levels in chronic lymphocytic leukemia are associated to low Myc and Skp2 expression, confer resistance to apoptosis and antagonize Myc effects on cell cycle
Source: Oncotarget. 2014 Jun 27;5(13):4694–708. doi: 10.18632/oncotarget.2100 (PMC4148092; doi:10.18632/oncotarget.2100)
Supplement: Supplementary file 1 [file oncotarget-05-4694-s001.pdf]

CARABALLO ET AL.

High p27 levels in chronic lymphocytic leukemia are associated to low Myc and Skp2 expression and confer resistance to apoptosis

SUPPLEMENTARY MATERIALS

Supplementary Table S1. Patient characteristics. 83 patient samples were analyzed at the RNA level, 107 at the protein level and 31 at both RNA and protein levels.

Characteristic	Value
**Sex**	
Female	33% (53)
Male	67% (106)
**Age at diagnosis (years)**	
Median	69,5 y.o.
Range	31-92 y.o.
**Stage (Rai)**	
0	43%
1	17%
2	19,5%
3	11,5%
4	9%
**Karyotype**	
Normal	43,5%
Abnormal	56,5%
**Treated patients**	52%
**Patients studied**	
p27 mRNA / protein	67 / 107
Myc mRNA / protein	83 / 102

Supplementary Table S2. Clinical characteristics of the patients overexpressing Myc protein. Rai, Rai stage at the sampling time point. Treat, treatment required at the time of the sampling. Prog., Progression of the disease is as defined in Materials and Methods. WC, white cell count at diagnosis.; DT, duplication time (DT): 0, <12 months; 1, >12 months, NK, not known. p27 and Myc protein levels are fold change versus controls, as defined in Materials and Methods;

Patient	Age	Rai	Treat.	Progr.	WC	DT	p27	Myc
01	60	3	No	No	10,500	0	2,80	1.02
02	66	0	No	No	12260	0	3,42	1,65
03	66	0	No	No	14,700	0	0.22	1.05
06	62	1	Yes	Yes	22,100	NK	6,69	1.64
11	69	1	Yes	No	3,990	0	4,42	1.10
14	76	0	No	No	12,900	0	4,97	1.39
16	50	4	Yes	Yes	9,020	NK	0,76	1.41
19	51	3	Yes	Yes	48,600	0	0,24	1.56
26	68	0	No	No	17,500	1	7.40	1.77
50	48	2	Yes	Yes	23,400	0	4.80	1.59
53	47	4	Yes	Yes	53,000	1	3.27	1.20
59	66	0	No	No	21,900	1	2.17	2.69
60	89	4	Yes	Yes	27,300	0	0.74	2.23
61	62	0	No	No	17,600	1	0	1.79
70	82	0	No	No	17,800	0	3.11	2.14
86	51	3	Yes	Yes	5,500	0	0	1.49
92	63	0	No	No	8,900	1	0	1.22
97	69	4	Yes	Yes	66,000	1	0	1.04
98	78	0	No	No	26,600	0	0	3.05

SUPPLEMENTARY FIGURES

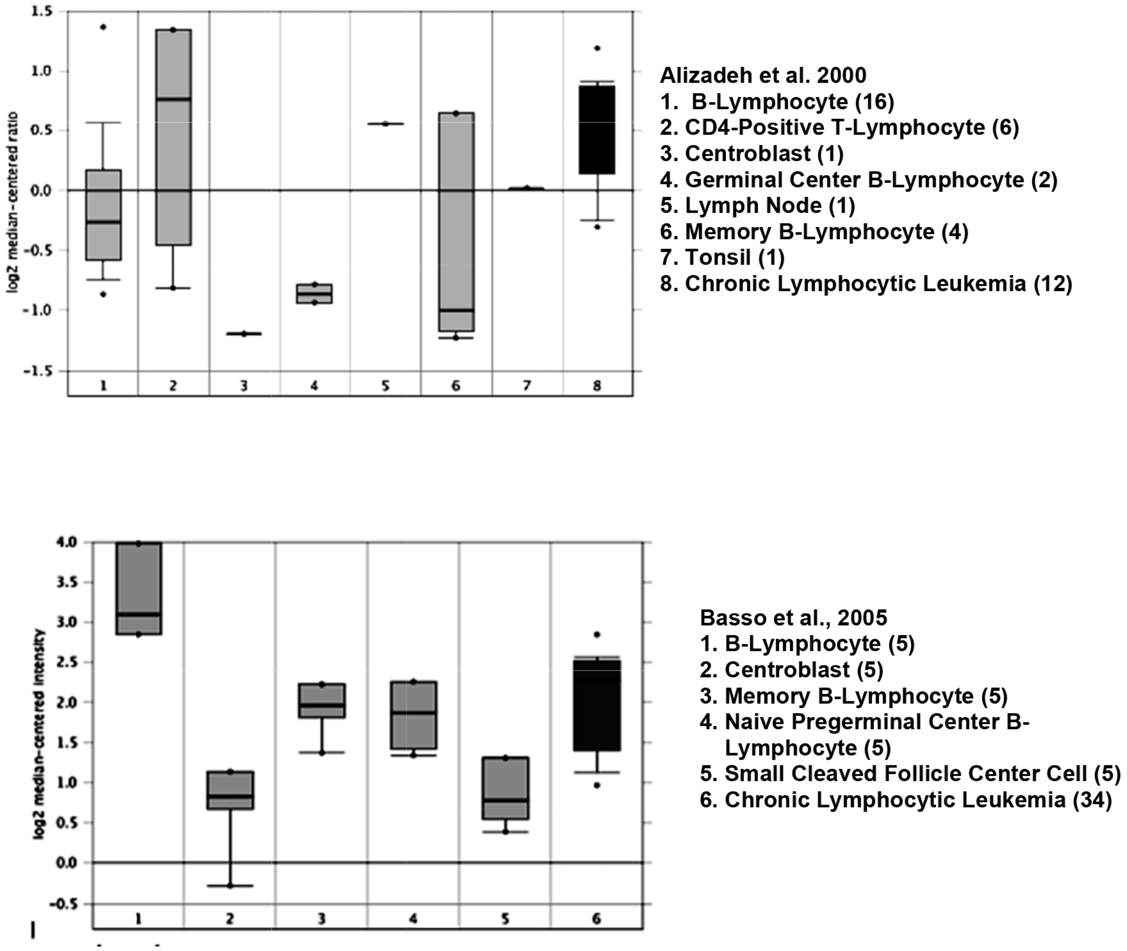

Supplementary Figure S1: Expression of p27 (CDKN1B) mRNA two different microarray-based studies from the Oncomine database are shown (Oncomine™, Compendia Bioscience, Ann Arbor, MI, https://www.oncomine.org/) (1, 2). The box plots shows the expression of p27 mRNA in different lymphoid cell controls and CLL (black box).

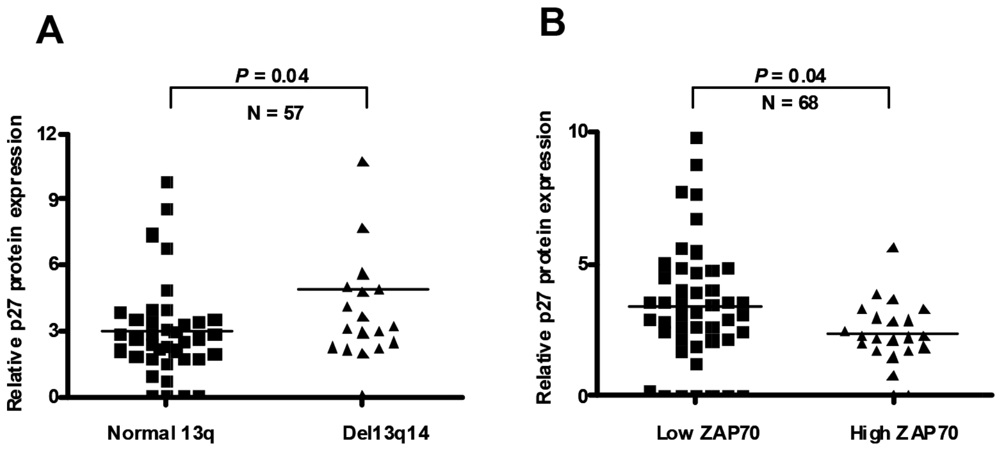

Supplementary Figure S2: Correlation between p27 protein expression with 13q14 deletion (A) and ZAP70 levels (B).

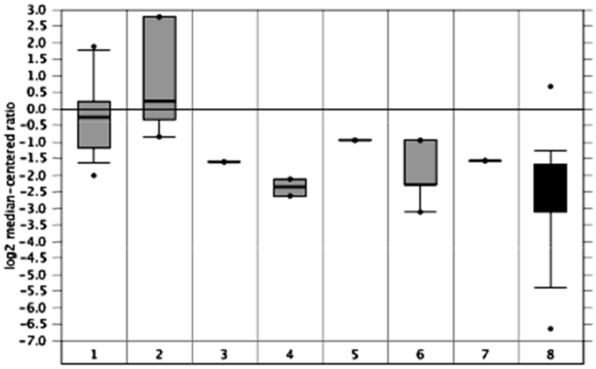

Alizadeh et al, 2000

1. B-Lymphocyte (16)
2. CD4-Positive T-Lymphocyte (6)
3. Centroblast (1)
4. Germinal Center B-Lymphocyte (2)
5. Lymph Node (1)
6. Memory B-Lymphocyte (4)
7. Tonsil (1)
8. Chronic Lymphocytic Leukemia (12)

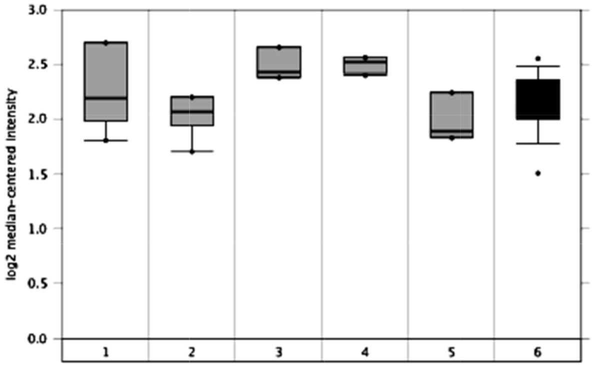

Basso et al., 2005

1. B-Lymphocyte (5)
2. Centroblast (5)
3. Memory B-Lymphocyte (5)
4. Naive Pregerminal Center B-Lymphocyte (5)
5. Small Cleaved Follicle Center Cell (5)
6. Chronic Lymphocytic Leukemia (34)

Probe 1827_s_at

Supplementary Figure S3: Expresion of Myc mRNA in two sets of leukemia samples. Two different microarray-based studies from the Oncomine database are shown (Oncomine™, Compendia Bioscience, Ann Arbor, MI, https://www.oncomine.org/). The box plots depict the differential expression of Myc. The box plots shows the expression of Myc mRNA in different lymphoid cell controls and CLL (black box).

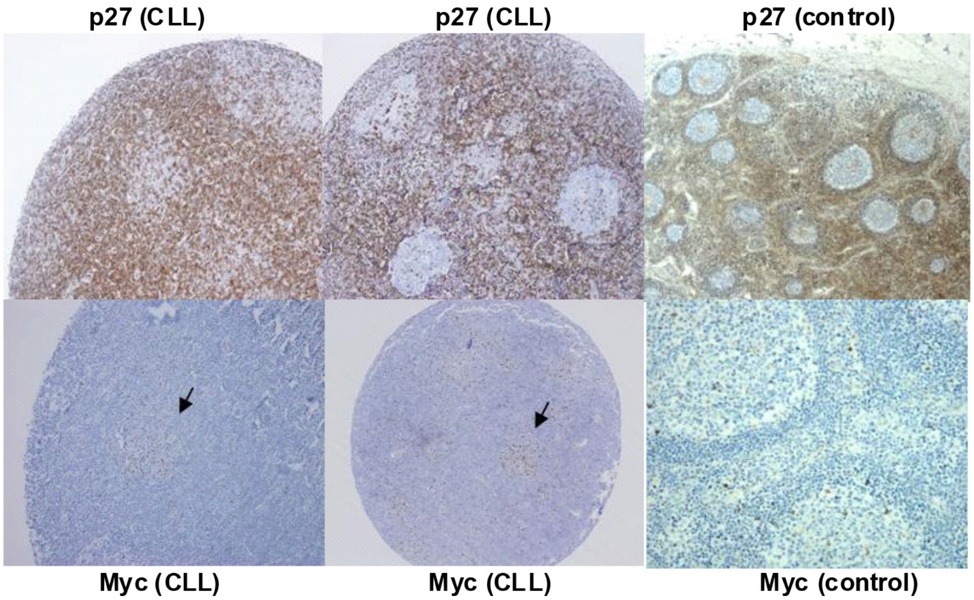

Supplementary Figure S4: Expression of p27 and Myc in representative CLL and healthy lymph nodes (control). Preparations of the nodes were subjected to immunohistochemistry for anti-Myc and anti-p27 antibodies as indicated in each figure. The arrows mark Myc-positive cells in proliferative centers of the nodes.

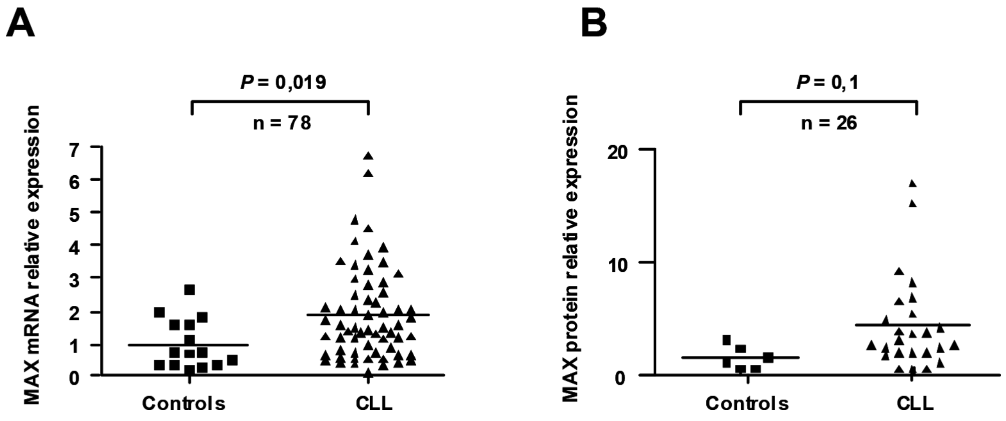

Supplementary Figure S5: Max expression in CLL. Expression of Max mRNA (A) and Max protein (B) in control (peripheral blood lymphocytes and tonsils) and CLL samples.

REFERENCES

1. Basso K, Margolin AA, Stolovitzky G, et al. Reverse engineering of regulatory networks in human B cells. Nat Genet. 2005; 37: 382-90.
2. Alizadeh AA, Eisen MB, Davis RE, et al. Distinct types of diffuse large B-cell lymphoma identified by gene expression profiling. Nature. 2000; 403: 503-11.
